# Modified endoscopic purse-string suture with dental floss traction in the management of a duodenal defect

**DOI:** 10.1055/a-2590-8450

**Published:** 2025-05-26

**Authors:** Bo Li, Silin Huang, Suhuan Liao, Miao He, Yin Xiao, Erzhen Zhong, Longbin Huang

**Affiliations:** 1 Department of Gastroenterology, South China Hospital, Medical School, Shenzhen University, Shenzhen, Guangdong, China

A 44-year-old man was admitted for endoscopic resection for a subepithelial lesion in the duodenal bulb during a routine health examination ( Fig. 1 **a** ). Endoscopic ultrasound revealed an exophytic hypoechoic lesion originating from the muscularis propria, with predominantly extraluminal growth, approximately 15 mm in diameter ( Fig. 1 **b** ). A contrast-enhanced abdominal CT scan was performed to exclude organs and lymph nodes metastasis. Following detailed communication with the patient, endoscopic submucosal excavation was undertaken. Postoperatively, only a minimal amount of serosal layer remained ( Fig. 2 **a** ). The narrow lumen of the duodenal bulb and the high tension of the mucosa posed a significant challenge for suturing the defect.To overcome this, we employed a modified purse-string suture technique ( Video 1 ). We carefully attached dental floss to each metal clip ( Fig. 2 **b** ), secured the nylon string to the edge of the defect, and gently pulled the dental floss. This technique effectively prevented the clips from inverting into the wound during the tightening ( Fig. 2 **c** and **d** ). Postoperatively, the patient remained fasting for 48 hours and was discharged on the fourth postoperative day without any complications such as fever or abdominal pain. Histopathological analysis confirmed the diagnosis of a gastrointestinal stromal tumor with a very low risk of malignancy ( Fig. 2 **e** and **f** ).

**Fig. 1:**
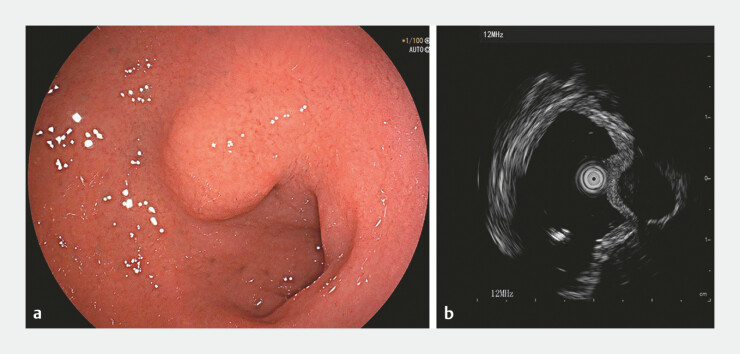
**a** White light endoscope showed a subepithelial lesion in the duodenal bulb measuring approximately 15 mm. **b** Endoscopic ultrasound revealed an exophytic hypoechoic lesion originating from the muscularis propria, with predominantly extraluminal growth.

**Fig. 2:**
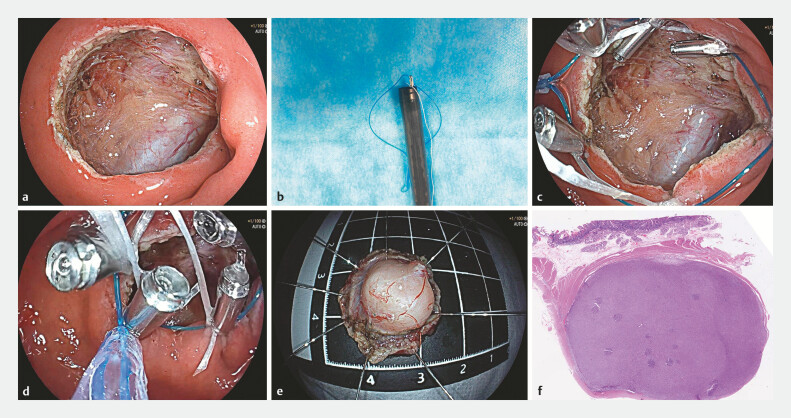
**a** Postoperative trauma. **b** Securing dental floss to each metal clip. **c** Clips with dental floss were deployed to anchor the edge of the defect. **d** Dental floss traction precluded the clip inverting into the wound during the tightening process. **e** Resected tumor. **f** Histopathological analysis confirmed a gastrointestinal mesenchymal tumor with a very low risk.

Modified endoscopic purse-string suture with dental floss traction has been effectively utilized in the management of duodenal defects.Video 1

The purse-string suture technique has proven effective in closing gastrointestinal tract defects, especially in challenging anatomical areas 1 2 . The incorporation of dental floss traction significantly enhances the efficiency, safety and resection rate in endoscopic surgeries 3 4 . In contrast to the conventional purse-string suture technique, our innovative approach, characterized by dental floss traction, mitigates the risk of inadvertent inversion of clips into the wound during nylon tightening ( Fig. 3 ). This innovation preserves the sutureʼs integrity and facilitates effective defect closure. To the best of our knowledge, this is the first report to describe the use of dental floss traction with metal clips in a purse-string suture for duodenal defect management, offering new insights into clinical endoscopic suture techniques.

**Fig. 3:**
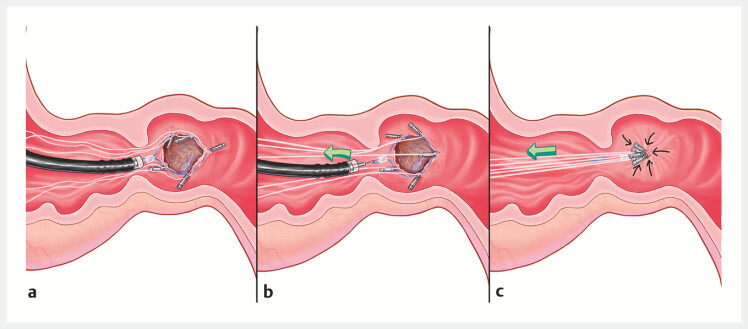
Illustration of modified endoscopic purse-string suture with dental floss traction.

Endoscopy_UCTN_Code_TTT_1AO_2AO

## Notes

Endoscopy E-Videos https://eref.thieme.de/e-videos
*E-Videos* is an open access online section of the journal *Endoscopy* , reporting on interesting cases and new techniques in gastroenterological endoscopy. All papers include a high-quality video and are published with a Creative Commons CC-BY license. Endoscopy E-Videos qualify for HINARI discounts and waivers and eligibility is automatically checked during the submission process. We grant 100% waivers to articles whose corresponding authors are based in Group A countries and 50% waivers to those who are based in Group B countries as classified by Research4Life (see: https://www.research4life.org/access/eligibility/ ). This section has its own submission website at https://mc.manuscriptcentral.com/e-videos .
